# Equity impacts of interventions to increase physical activity among older adults: a quantitative health impact assessment

**DOI:** 10.1186/s12966-020-00999-4

**Published:** 2020-08-14

**Authors:** Johanna-Katharina Schönbach, Gabriele Bolte, Gesa Czwikla, Kristin Manz, Monika Mensing, Saskia Muellmann, Claudia Voelcker-Rehage, Stefan K. Lhachimi

**Affiliations:** 1 University of Bremen, Institute of Public Health and Nursing Research, Department of Social Epidemiology, Bremen, Germany; 2 University of Bremen, Health Sciences Bremen, Bremen, Germany; 3 Robert Koch Institute, Berlin, Germany; 4 NRW Centre for Health, Bochum, Germany; 5 Leibniz Institute for Prevention Research and Epidemiology – BIPS, Bremen, Germany; 6 Chemnitz University of Technology, Institute of Human Movement Science and Health, Chemnitz, Germany; 7 University of Münster, Institute of Sport and Exercise Sciences, Münster, Germany; 8 University of Bremen, Institute of Public Health and Nursing Research, Department of Health Services Research, Bremen, Germany

**Keywords:** Health impact assessment, Physical activity, Social inequalities, Interventions, Older adults

## Abstract

**Background:**

Behavioural interventions may increase social inequalities in health. This study aimed to project the equity impact of physical activity interventions that have differential effectiveness across education groups on the long-term health inequalities by education and gender among older adults in Germany.

**Methods:**

We created six intervention scenarios targeting the elderly population: Scenarios #1–#4 applied realistic intervention effects that varied by education (low, medium high). Under scenario #5, all older adults adapted the physical activity pattern of those with a high education. Under scenario #6, all increased their physical activity level to the recommended 300 min weekly. The number of incident ischemic heart disease, stroke and diabetes cases as well as deaths from all causes under each of these six intervention scenarios was simulated for males and females over a 10-year projection period using the DYNAMO-HIA tool. Results were compared against a reference-scenario with unchanged physical activity.

**Results:**

Under scenarios #1–#4, approximately 3589–5829 incident disease cases and 6248–10,320 deaths could be avoided among males over a 10-year projection period, as well as 4381–7163 disease cases and 6914–12,605 deaths among females. The highest reduction for males would be achieved under scenario #4, under which the intervention is most effective for those with a high education level. Scenario #4 realizes 2.7 and 2.4% of the prevented disease cases and deaths observed under scenario #6, while increasing inequalities between education groups. In females, the highest reduction would be achieved under scenario #3, under which the intervention is most effective amongst those with low levels of education. This scenario realizes 2.7 and 2.9% of the prevented disease cases and deaths under scenario #6, while decreasing inequalities between education groups. Under scenario #5, approximately 31,687 incident disease cases and 59,068 deaths could be prevented among males over a 10-year projection period, as well as 59,173 incident disease cases and 121,689 deaths among females. This translates to 14.4 and 22.2% of the prevented diseases cases among males and females under scenario #6, and 13.7 and 27.7% of the prevented deaths under scenario #6.

**Conclusions:**

This study shows how the overall population health impact varies depending on how the intervention-induced physical activity change differs across education groups. For decision-makers, both the assessment of health impacts overall as well as within a population is relevant as interventions with the greatest population health gain might be accompanied by an unintended increase in health inequalities.

## Background

Physical activity is a major contributor to healthy ageing [1, 2]. For instance, being sufficiently physically active has been shown to play an important role in the prevention of various non-communicable diseases frequently occurring in the older population such as cardiovascular diseases, diabetes and cancer [3–5]. The World Health Organization (WHO) therefore recommends adults aged 65 years and above do a minimum of 150 min of moderate-intensity physical activity or 75 min of vigorous physical activity per week in addition to muscle-strengthening activities. For additional health benefits, total weekly physical activity should be increased to 300 min of moderate-intensity, or 150 min of vigorous-intensity [6]. However, only 59% of the European population over the age of 65 years achieve the recommended minimum level [7].

Notable differences also exist between population groups characterised by gender or by education, which is a widely recognised indicator of socioeconomic position [8]. In particular, recreational physical activity in elderly men is higher than in women, whereas domestic and garden-related physical activity in elderly women is higher than in men [9]. At the same time, non-work-related physical activity among older people with higher education (commonly defined as completed tertiary education) is more common than among those with low education (commonly defined as completed lower secondary school at most) [9]. Among adults aged 65 years and above living in Germany, for example, 51% of women and 55% of men with higher education achieve at least 150 min of physical activity in their leisure time and with cycling for transport, compared to 29% of women and 36% of men with low education levels [10]. Because regular physical activity is a major determinant of health, these low levels of health-enhancing physical activity among less educated individuals are considered an important contributor to inequalities in health [11–13]. In Germany, the prevalence of different diseases, such as ischemic heart disease (IHD) [14], stroke [15] and diabetes [16], is higher among people with low levels of education than among those with higher education [17]. Educational attainment may be relevant in this context because it can provide material and psychological resources for a healthy lifestyle [18]. For instance, education conveys knowledge as well as cognitive and emotional skills, which are important conditions that could enable adults to be more receptive to educational messages, modify risk behaviours and make healthy decisions over their life course. In addition to that, these skills may facilitate the communication and access to appropriate health services [19, 20]. Furthermore, education is a strong determinant of future employment and income [20], and it may lead to social capital and a social network that provides advantages [21]. Given the high prevalence of insufficient physical activity in populations, the WHO considers physical activity to be an important target for health promotion [2]. In its Global Action Plan, the WHO sets out to achieve a 15% reduction in physical inactivity by 2030 [22]. Synthesized evidence, often from randomized controlled trials [23, 24], provides an indication of what changes in physical activity can be expected from interventions. From these trials, however, it remains unclear what population level health effects can be expected from intervention-related changes in physical activity given differences in the age structure and incidence-prevalence-mortality profile of chronic diseases such as IHD, stroke and diabetes across populations. Previous research has also found that many of these studies do not examine potentially differential intervention effects by social characteristics, such as educational attainment [25, 26].

It is particularly important that interventions are evaluated for different population groups as interventions that might be successful in a population overall may inadvertently increase health inequalities in particular groups. This is the case if socioeconomically advantaged groups benefit more from an intervention than disadvantaged groups. These inequalities may arise during different stages of an intervention and can relate to access, uptake, and coherence [27]. Interventions that rely on voluntary behaviour change can be particularly difficult in this regard as such interventions usually require considerable cognitive, psychological, time, and material resources that socioeconomically advantaged individuals tend to have more of [28].

Health impact assessments systematically predict the future health impact of an intervention and its distribution within a specific population. By quantifying the overall effects of interventions for a particular context, health impact assessments enable policy makers to compare options and to make optimal decisions for a specific population of interest [29]. Although health equity is considered a core element of health impact assessments [30, 31], many quantitative assessments of intervention impacts on population health do not explicitly analyse potential differential impacts on population subgroups and their meaning for health inequities [32–34].

In this study, we conducted a health impact assessment of physical activity interventions that quantified both the long-term health gains and health inequalities if applied to the whole population of older adults in Germany. In particular, we examined how the effectiveness of the interventions may influence health disparities between females and males given different levels of educational attainment.

## Methods

### Context

This health impact assessment was conducted within the project “EQUAL – Equity impacts of interventions to increase physical activity” [35], which is a subproject of the research network “AEQUIPA – Physical activity and health equity: primary prevention for healthy ageing” [36].

The intervention effect estimates stem from the project “PROMOTE – Tailoring physical activity interventions to promote healthy ageing” [37], which is also a subproject of the AEQUIPA research network. Intervention scenarios were created to reflect typical policy choices.

### Intervention scenarios

To model the health impacts of differences in the effectiveness of a physical activity intervention across social characteristics, we used the results of the PROMOTE project as a case study and also applied a range of hypothetical intervention scenarios.

PROMOTE consisted of two web-based interventions and a delayed intervention control group conducted among 589 adults aged 65 to 79 years in five German communities. Intervention group 1 received access to a web-based intervention for self-tracking physical activity behaviour. Intervention group 2 additionally received an activity tracker [37]. Re-analysis of the intervention effects was done according to a previously defined strategy within the EQUAL project [38]. This found the accelerometer-measured weekly moderate-to-vigorous physical activity (MVPA) in both intervention groups combined was 7.59 min higher than in the control group 12 weeks after baseline (end of intervention) after adjusting for baseline physical activity, community, valid wear-time, season, age, gender and education level (95% CI: 2.58; 12.61; *n* = 350). This effect was modelled in our scenario #1 *(PROMOTE-undifferentiated)*. When taking effect modification by education into account, MVPA in the intervention group was 19.70 min higher (95% CI: − 18.7; 58.15; *n* = 6) compared to the control group among those with low education, 5.80 min higher (95% CI: − 1.37; 12.97; *n* = 168) among those with medium education and 9.53 min higher (95% CI: 2.30; 16.76; *n* = 176) among those with high education (model adjusted for baseline physical activity, community, valid wear-time, season, age, gender and possible interaction of intervention group and gender). Although the stratified education effects were not statistically significant at the 95% level among the medium educated and low educated groups, we used these unbiased point estimates as pragmatic parameter estimates [39, 40] for our scenario #2 *(PROMOTE-differentiated)*. Due to the small sample size, the education-specific effect estimates from a re-analysis of the PROMOTE project could not be further differentiated by gender.

In addition to the PROMOTE case study, we modelled two scenarios in which intervention effects showed a different, fictitious gradient across education groups. In scenario #3 *(Downward gradient)*, we modelled an intervention that is most effective in people with low education and least effective in people with high education. Scenario #3 may therefore represent interventions regarded as “universal policy with additional focus on gap” or “proportionate universalism” [41]. Interventions of these types benefit the whole population, but focus specifically on the most socioeconomically disadvantaged population groups [41]. A reversed gradient with the least effectiveness in people with low education and the greatest effectiveness in those with high education was illustrated in scenario #4 *(Upward gradient)*. Scenario #4 may be an example of a “potential increase in the variation of risk following a population-approach” intervention, in which those at lower risk exposure gain more benefits from the intervention than those at greater risk exposure [42]. Finally, we modelled two scenarios that completely eliminated inequality in physical activity between education groups. Scenario #5 *(Equalize)* assumed that people with low and medium levels of education adapted the same physical activity patterns as those with high education. In scenario #6 *(Guideline)* we assumed that all education groups increased their physical activity to the recommended 300 min per week. This scenario served as the most desirable policy goal and presented the maximum achievable health gain. An overview of scenarios is presented in Table 1.

**Table 1:** Physical activity change in people aged ≥55 years across scenarios

Name of scenario	Description of intervention-induced change in MVPA-minutes (and corresponding MET-hours^**a**^) per week
#Reference-scenario	Physical activity remains at the currently observed level
#1: PROMOTE-undifferentiated	+ 7.59 MVPA-minutes (+ 0.76 MET-hours) per week, for older adults with low, medium or high education
#2: PROMOTE-differentiated	+ 19.70 MVPA-minutes (+ 1.97 MET-hours) per week for older adults with low education, + 5.80 MVPA-minutes (+ 0.58 MET-hours) per week for older adults with medium education, + 9.53 MVPA-minutes (+ 0.95 MET-hours) per week for older adults with high education
#3: Downward-gradient	+ 19.70 MVPA-minutes (+ 1.97 MET-hours) per week for older adults with low education, + 9.53 MVPA-minutes (+ 0.95 MET-hours) per week for older adults with medium education, + 5.80 MVPA-minutes (+ 0.58 MET-hours) per week for older adults with high education
#4: Upward-gradient	+ 5.80 MVPA-minutes (+ 0.58 MET-hours) per week for older adults with low education, + 9.53 MVPA-minutes (+ 0.95 MET-hours) per week for older adults with medium education, + 19.70 MVPA-minutes (+ 1.97 MET-hours) per week for older adults with high education
#5: Equalizing	All older adults with low or medium education adapt the physical activity profile of older adults with high education
#6: Guideline	All older adults increase their physical activity level to ≥300 MVPA-minutes (≥22.50 MET-hours) per week

^a^ assuming 6 metabolic equivalents of tasks (METs) for activities in moderate-to-vigorous physical activity (MVPA) intensity, being the intersection of moderate-intensity activities with an energy expenditure of 3–5.9 METs, and vigorous-intensity with ≥6 METs [43, 44]. Exemplary calculation: 7.59 MVPA-minutes*6METs/60 min = 0.759 MET-hours

In order to account for the intensity of activities (e.g. moderate, moderate-to-vigorous, vigorous), we converted the intervention effect from minutes in MVPA to the metabolic equivalent of task (MET)-hours per week (MET-hours). For this purpose, we multiplied the change in MVPA-minutes by the corresponding MET-value. MET values reflect the energy expenditure of specific activities and are expressed as multiples of the energy cost of sitting at rest, which is equivalent to 1 MET [43, 44]. Activities of moderate-intensity are defined as 3–5.9 METs, while the energy expenditure required to perform vigorous-intensity activities are ≥6 METs [43, 44]. We assigned MVPA a MET-value of 6, which is the intersection of both categories.

In all scenarios, we assumed that the intervention-induced physical activity change would occur immediately and population-wide in all people aged 55 and over, i.e. in the period of transition to retirement [24]. We assumed that the new physical activity pattern of this cohort would remain stable over the projection period (implemented in the DYNAMO-HIA software tool as zero transitions).

### Physical activity over scenarios

We determined current physical activity levels from the German Health Update 2014/2015 (GEDA 2014/2015-EHIS) dataset [45]. The GEDA 2014/2015-EHIS study was conducted by the Robert Koch Institute from November 2014 to July 2015. This study collected data from 24,016 adults aged 18 and above [10, 46]. It implemented the questionnaire of the European Health Interview Survey (EHIS), which was coordinated by Eurostat and aimed to provide harmonized and comparable data not only on health determinants but also on health status, health care use and socioeconomic background variables for European countries [46, 47]. To assess physical activity, the German version of the European Health Interview Survey-Physical Activity Questionnaire (EHIS-PAQ) was applied [48, 49]. In the questionnaire, participants were asked how many days a week they were walking or biking for transportation, respectively. If they stated they walk or bike at least once a week, they were asked to indicate the duration per day (10 to 29 min, 30 to 59 min, 1 to < 2 h, 2 to < 3 h, ≥3 h per day). Participants were also asked how many days a week they were doing physical activity in their leisure time (excluding transport related activities). If they stated that they were doing this at least once a week, they were asked to report the number of hours and minutes per week. We converted these minutes and hours spent in the differing physical activity domains to MET-hours per week. We assigned ‘Walking’ a MET-value of 3.3 [44], ‘Biking’ a MET-value of 6 [44] and ‘Sport’ a MET-value of 4.5 (the midpoint of moderate-intensity defined with 3–5.9 METs).

For each of the 22,354 participants with complete information on walking, biking and leisure time physical activity, the weekly minutes of all three domains were added together to create a combined variable of total physical activity in MET-hours per week.

The level of education in GEDA2014/2015-EHIS was based on the international standard classification of education (ISCED) [50]. GEDA2014/2015-EHIS categorizes education as ‘low’ (ISCED levels 0 to 2, i.e. early childhood education, primary education and lower secondary education), ‘medium’ (ISCED levels 3 to 4, i.e. upper secondary education and post-secondary non-tertiary education) and ‘high’ (ISCED levels 5 to 8, i.e. short-cycle tertiary education, Bachelor or equivalent level, Masters or equivalent level, or Doctoral or equivalent level) [51]. We fitted the individual-level physical activity data by gender, seven age groups (18–24, 25–34, 35–44, 45–54, 55–64, 65–69, 70+ years) and three education groups (low, medium, high) to a Weibull distribution. Using the Weibull’s shape and scale parameter from the respective fit, we calculated the mean physical activity by gender, age and education of the reference-scenario. For the intervention scenarios, we shifted the mean according to the intervention effect (see Table 1).

For all scenarios, we estimated the proportion of people falling into three categories by gender, age and education: insufficiently active (0 < 11.25 MET-hours/week), sufficiently active (≥11.25 < 22.5 MET-hours/week) and additionally active (≥22.5 MET-hours/week) (Table 2).

**Table 2:** Categorization of physical activity levels

Category	Physical activity in moderate-intensity (with corresponding MET-hours) per week^**a**^
Insufficiently active	< 150 min of physical activity in moderate-intensity (corresponding to < 11.25 MET-hours) per week
Sufficiently active	≥150 < 300 min of physical activity in moderate-intensity (corresponding to ≥11.25 < 22.5 MET-hours) per week
Additionally active	≥300 min of physical activity in moderate-intensity (corresponding to ≥22.5 MET-hours) per week

^a^ assuming 4.5 metabolic equivalents of tasks (METs) since moderate-intensity activities require an energy expenditure of 3–5.9 METs [43, 44]

### Relative risks

Associations between levels of physical activity and IHD, stroke, diabetes as well as all-cause mortality were identified from the literature. For IHD, stroke and diabetes, we used a meta-analysis that reported relative risks for an increase of 11.25 MET-hours/week, under the assumption that the relationship followed a 0.25 power transformation [52]. The relative risks were based on studies in which physical activity included at least two out of the four domains of leisure, household, active travel, and occupational activity.

For all-cause mortality, we used relative risks from a published pooled analysis that reported the relative risk for seven categories of MET-hours per week. In the underlying studies, physical activity included walking, jogging or running, swimming, tennis, bicycling, aerobics, and dance [53].

The relative risks for IHD, stroke, diabetes as well as all-cause mortality are shown in Table 3. We applied the same relative risks to all three education groups.

**Table 3:** Relative risks for IHD, stroke, diabetes and all-cause mortality

Outcome	Original RRs as presented in publications	Transformed relative risks		
		0 < 11.25 MET-hours/week	≥11.25 < 22.5 MET-hours/week	≥22.5 MET-hours/week
**IHD**	11.25 MET-hours/week increase: RR = 0.77 [52] ^1^	1.00 (m, f)	0.77 (m, f)	0.73 (m, f)
**Stroke**	11.25 MET-hours/week increase: RR = 0.78 [52] ^1^	1.00 (m, f)	0.78 (m, f)	0.74 (m, f)
**Diabetes**	11.25 MET-hours/week increase: RR = 0.73 [52] ^1^	1.00 (m, f)	0.73 (m, f)	0.68 (m, f)
**All-cause mortality**	0 MET-hours/week: RR = 1 (m, f); 0.1 < 7.5 MET-hours/week: RR = 0.82 (m), RR = 0.77 (f); 7.5 < 15 MET-hours/week: RR = 0.71 (m), 0.67 (f); 15 < 22.5 MET-hours/week: RR = 0.63 (m), RR = 0.64 (f); 22.5 < 40 MET-hours/week: RR 0.61 (m), RR = 0.60 (f); [53] ^2^	1.00 (m, f)	0.70 (m), 0.68 (f)	0.61 (m), 0.64 (f)

*IHD* Ischemic heart disease; *MET* Metabolic equivalent of task; *m* Males; *f* Females; *RR* Relative risks.

^1^ All of the studies underlying the meta-analysis are adjusted for multiple potential confounding variables, but not all confounders were adjusted for in every study; the estimate from meta-analysis was unadjusted for BMI

^2^ Adjusted for smoking, alcohol, education, marital status, history of cancer, history of heart disease and BMI

### Population and disease data

Data on Germany’s population size, age-composition, projected births and mortality as well as disease incidence, prevalence and mortality for IHD, stroke and type 2 diabetes were derived from the DYNAMO-HIA database, which is publicly available from the DYNAMO-HIA website [54]. The data is provided by gender for each age year from 0 to 95, as needed for the health impact assessment.

### Dynamic modelling

In order to quantify the health impacts of the respective changes in physical activity, we used DYNAMO-HIA. DYNAMO-HIA is a software tool to conduct quantitative health impact assessments [55, 56] that is freely available from the website [54] and has previously been used for other health impact assessments [57–64].

In this health impact assessment, the DYNAMO-HIA software tool projected the six intervention scenarios in comparison to the reference-scenario over a projection period of 10 years. This projection period is the recommended time span [65] and is in line with previous health impact assessments [60, 63, 64].

DYNAMO-HIA first classified the simulated individuals to a physical activity category for every year in the simulation. Each individual was then assigned the probability of having a disease and the probability of being alive [56]. At the end of the simulation, disease incidences and deaths from all causes for males and females were compared between the scenarios.

## Results

We present the results for each scenario in turn. The overall and education-specific differences between each of the six intervention scenarios in comparison to the reference-scenario are shown in Table 4 for the cumulated number of incident IHD, stroke and diabetes cases and in Table 5 for deaths from all causes.

**Table 4:** Incident IHD, stroke and diabetes cases, cumulated over the 10-year projection period (among people aged ≥55 years in projection year 1)

	Males				Females			
Scenarios	Overall	Low education	Medium education	High education	Overall	Low education	Medium education	High education
**Incident cases in the reference-scenario**								
#Ref: Reference-scenario	2,289,341	269,392	1,279,684	740,265	2,385,012	908,950	1,227,391	248,671
**Intervention-induced health effects (Difference in incident cases between intervention scenarios and reference-scenario)**								
#1: PROMOTE-undifferentiated	−3589	− 677	− 1694	− 1218	−4381	− 1893	− 1922	− 566
#2: PROMOTE-differentiated	− 3743	− 1045	− 1256	− 1442	− 6220	− 4334	− 1264	−622
#3: Downward-gradient	− 4172	−1045	− 2128	− 999	− 7163	−4334	− 2463	− 366
#4: Upward-gradient	−5829	− 555	− 2128	− 3146	− 4966	− 1531	−2463	− 972
#5: Equalizing	−31,687	− 8380	−23,307	0	− 59,173	−37,841	−21,332	0
#6: Guideline	−219,783	−29,969	− 127,878	− 61,936	−266,496	− 112,281	− 131,041	−23,174
**Distribution of intervention-induced health gain (Education-specific intervention effects measured against overall intervention effect)**								
#1: PROMOTE-undifferentiated	100%	18.9%	47.2%	33.9%	100%	43.2%	43.9%	12.9%
#2: PROMOTE-differentiated	100%	27.9%	33.6%	38.5%	100%	69.7%	20.3%	10.0%
#3: Downward-gradient	100%	25.0%	51.0%	23.9%	100%	60.5%	34.4%	5.1%
#4: Upward-gradient	100%	9.5%	36.5%	54.0%	100%	30.8%	49.6%	19.6%
#5: Equalizing	100%	26.4%	73.6%	0.0%	100%	63.9%	36.1%	0.0%
#6: Guideline	100%	13.6%	58.2%	28.2%	100%	42.1%	49.2%	8.7%
**Proportion of the maximal achievable health gain (Intervention-induced health effects in scenarios #1 to #5 measured against intervention-induced health effects in scenario #6)**								
#1: PROMOTE-undifferentiated	1.6%	2.3%	1.3%	2.0%	1.6%	1.7%	1.5%	2.4%
#2: PROMOTE-differentiated	1.7%	3.5%	1.0%	2.3%	2.3%	3.9%	1.0%	2.7%
#3: Downward-gradient	1.9%	3.5%	1.7%	1.6%	2.7%	3.9%	1.9%	1.6%
#4: Upward-gradient	2.7%	1.9%	1.7%	5.1%	1.9%	1.4%	1.9%	4.2%
#5: Equalizing	14.4%	28.0%	18.2%	0.0%	22.2%	33.7%	16.3%	0.0%
#6: Guideline	100.0%	100.0%	100.0%	100.0%	100.0%	100.0%	100.0%	100.0%

**Table 5:** Deaths from all causes cumulated over the 10-year projection period (among people aged ≥55 years in projection year 1)

	Males				Females			
Scenarios	Overall	Low education	Medium education	High education	Overall	Low education	Medium education	High education
**Deaths in the reference-scenario**								
#Ref: Reference-scenario	3,426,790	419,697	1,912,300	1,094,793	3,977,866	1,677,687	1,941,934	358,245
**Intervention-induced health effects (Difference in deaths between intervention scenarios and reference-scenario)**								
#1: PROMOTE-undifferentiated	−6248	− 1065	− 2962	− 2221	−6914	− 3452	− 2912	− 550
#2: PROMOTE-differentiated	− 6713	− 1771	− 2231	− 2711	−11,422	− 8678	− 2120	−624
#3: Downward-gradient	− 7437	−1771	− 3834	− 1832	−12,605	− 8678	− 3558	− 369
#4: Upward-gradient	−10,320	− 904	− 3834	− 5582	− 7187	− 2672	− 3558	− 957
#5: Equalizing	− 59,068	− 15,199	−43,869	0	− 121,689	− 85,603	− 36,086	0
#6: Guideline	−430,143	−59,414	− 249,121	− 121,608	− 439,722	− 213,319	− 195,575	−30,828
**Distribution of health benefits (Education-specific intervention effects measured against overall intervention effect)**								
#1: PROMOTE-undifferentiated	100%	17.0%	47.4%	35.5%	100%	49.9%	42.1%	8.0%
#2: PROMOTE-differentiated	100%	26.4%	33.2%	40.4%	100%	76.0%	18.6%	5.5%
#3: Downward-gradient	100%	23.8%	51.6%	24.6%	100%	68.8%	28.2%	2.9%
#4: Upward-gradient	100%	8.8%	37.2%	54.1%	100%	37.2%	49.5%	13.3%
#5: Equalizing	100%	25.7%	74.3%	0.0%	100%	70.3%	29.7%	0.0%
#6: Guideline	100%	13.8%	57.9%	28.3%	100%	48.5%	44.5%	7.0%
**Proportion of the maximal achievable health gain (Intervention effects in scenarios #1 to #5 measured against intervention effect in scenario #6)**								
#1: PROMOTE-undifferentiated	1.5%	1.8%	1.2%	1.8%	1.6%	1.6%	1.5%	1.8%
#2: PROMOTE-differentiated	1.6%	3.0%	0.9%	2.2%	2.6%	4.1%	1.1%	2.0%
#3: Downward-gradient	1.7%	3.0%	1.5%	1.5%	2.9%	4.1%	1.8%	1.2%
#4: Upward-gradient	2.4%	1.5%	1.5%	4.6%	1.6%	1.3%	1.8%	3.1%
#5: Equalizing	13.7%	25.6%	17.6%	0.0%	27.7%	40.1%	18.5%	0.0%
#6: Guideline	100.0%	100.0%	100.0%	100.0%	100.0%	100.0%	100.0%	100.0%

### Reference scenario

Summed over the 10-year projection period, 2,289,341 male and 2,385,012 female incident IHD, stroke and diabetes cases as well as 3,426,790 male and 3,977,866 female deaths are expected to occur under the reference scenario among those aged 55 years and older in projection year 1.

### Scenario #6 *(Guideline)*

Under scenario #6 *(Guideline)*, 219,783 male and 266,496 female incident disease cases as well as 430,143 male and 439,722 female deaths could be eliminated by moving all adults to the highest physical activity category.

### Scenarios #1 to #4: intervention-induced health effects in males

Among the first four scenarios, the smallest reduction compared to the reference scenario in males is expected to occur under scenario #1 *(PROMOTE-undifferentiated)*. Under scenario #1, there are 3589 fewer incident IHD, stroke and diabetes cases as well as 6248 fewer deaths. This is then followed by scenario #2 *(PROMOTE-differentiated)*, scenario #3 *(Downward-gradient) and* scenario #4 *(Upward-gradient)*. There are 5829 fewer incident disease cases and 10,320 fewer deaths in scenario #4 compared to the reference scenario.

### Scenarios #1 to #4: distribution of intervention-induced health gain in males

In two of these scenarios, #2 *(PROMOTE-differentiated)* and #4 *(Upward-gradient)*, the largest proportion of the overall health benefit occurs amongst males with high education, while the lowest proportion occurs amongst those with low education. In scenario #1 *(PROMOTE-undifferentiated)*, half of the overall health benefit is experienced by males with medium education, while one third is experienced by males with high education and one fifth is experienced by those with low education. In scenario #3 *(Downward-gradient)*, half of the health benefit is experienced by those with medium education and one quarter experienced by each of the low and high education groups, respectively.

### Scenarios #1 to #4: proportion of the maximal achievable health gain in males

The reduction of incident disease cases and the reduction of deaths in males under scenario #1 *(PROMOTE-undifferentiated)* corresponds to 1.6 and 1.5% of what could maximally be reduced under scenario #6 *(Guideline)*. This is followed by scenario #2 *(PROMOTE-differentiated)* and #3 *(Downward-gradient)*, up to scenario #4 *(Upward-gradient)*. Scenario #4 avoids 2.7% of the incident disease cases and 2.4% of the deaths that could maximally be reduced. With regard to education level, males with low levels of education achieve more, or at least as much, of the maximum achievable health gains than medium and high education groups under scenario #1 *(PROMOTE-undifferentiated)*, scenario #2 *(PROMOTE-differentiated)* and scenario #3 *(Downward-gradient)*. In scenario #4 *(Upward-gradient)*, males with higher levels of education experience more of the maximally achievable health gains than the low or medium education groups.

### Scenarios #1 to #4: intervention-induced health effects in females

For females, the smallest reduction among the first four scenarios compared to the reference-scenario is expected to occur under scenario #1 *(PROMOTE-undifferentiated)*. Under this scenario, there are 4381 fewer incident IHD, stroke and diabetes cases as well as 6914 fewer deaths. This is followed by scenario #4 *(Upward-gradient)*, scenario #2 *(PROMOTE-differentiated)* and then scenario #3 *(Downward-gradient)*, under which there are 7163 fewer disease cases and 12,605 fewer deaths.

### Scenarios #1 to #4: distribution of intervention-induced health gain in females

Across all four scenarios, the smallest share of health benefit is experienced by those with high education. In scenarios #2 *(PROMOTE-differentiated)* and #3 *(Downward-gradient),* approximately two thirds of the overall health benefit is experienced by females with low education and not more than 10% by those with high education. In scenario #4 *(Upward-gradient)*, half of the health benefit is experienced by females with medium education and one third by those with low education. In scenario #1 *(PROMOTE-undifferentiated)*, more than 40% is experienced by females with low and medium education, each.

### Scenarios #1 to #4: proportion of the maximal achievable health gain in females

Among females, the reduction of incident disease cases and the reduction of deaths under each of the first four scenarios corresponds to 1.6% of what could maximally be reduced under scenario #6 *(Guideline)*. This is followed by scenario #4 *(Upward-gradient)* and #2 *(PROMOTE-differentiated)* and then scenario #3 *(Downward-gradient)*, which avoids 2.7 and 2.9% of the maximum number of reducible incident disease cases and deaths. In scenario #2 *(PROMOTE-differentiated)* and #3 *(Downward-gradient)*, females with low levels of education achieve more of the maximal achievable health gains than medium or highly educated females. Scenario #1 *(PROMOTE-undifferentiated)* and #4 *(Upward-gradient)* realize more of the maximally achievable health gains for highly educated females than for low and medium educated.

### Scenario #5 *(Equalizing)*

If people with low and medium education adapted the physical pattern of people with high education as simulated in scenario #5 *(Equalizing)*, the number of incident IHD, stroke and disease cases would be reduced by 31,687 in males and 59,173 in females. Moreover, the number of deaths would be reduced by 59,068 and 121, 689, respectively. This corresponds to 14.4 and 22.2% of the maximal reducible incident disease cases as shown in scenario #6 *(Guideline)*, as well as 13.7 and 27.7% of the maximal reducible number of respective deaths among males and females.

## Discussion

### Discussion of the main findings

We used the DYNAMO-HIA software tool to model the long-term population health effects from physical activity interventions. We applied our model to the whole population aged ≥55 years in Germany, and examined how differential effectiveness across education groups impact health disparities.

Presuming a similar physical activity change in all education groups, as in scenario #1 *(PROMOTE-undifferentiated)*, our results showed that approximately 3589 disease cases and 6248 deaths among males as well as 4381 disease cases and 6914 deaths in females could be saved over a 10-year projection period. The overall population health effects for males do not change in a substantial way when differential effectiveness across education groups is taken into account as in scenario #2 *(PROMOTE-differentiated)*. Amongst females, however, the overall population health benefit would increase substantially with 6220 averted disease cases and 11,422 avoided deaths. Thus, our results emphasize that the evaluation of education-specific intervention effects is crucial for assessing the magnitude of the exact population health impact. This finding is important, since previous research has highlighted the fact that studies on health impact assessments of physical activity interventions often do not examine how intervention effects can differ by social characteristics such as education [25, 26].

Among males, an intervention with a gradient as in scenario #4 *(Upward-gradient)* would have a bigger overall health benefit than scenarios #1 *(PROMOTE-undifferentiated)*, #2 *(PROMOTE-differentiated)* and #*3 (Downward-gradient)*. Scenario #4 is an example of intervention-generated inequalities as it is the most effective among those with high education and so would result in an increase of health inequalities between education groups [42, 66]. The other three scenarios, however, would result in a decrease of health inequalities between education groups. Thus, in our simulation and its underlying assumptions, there appears to be a balancing act between increasing overall population health and increasing inequalities in males. Obviously, comprehensive equity-focused health impact assessments are necessary as a basis for informed decision-making in public health with its main challenge of reducing health inequities [42].

Among females, the most disease cases and deaths would be prevented in scenario #3 *(Downward-gradient)*. Under this scenario, the intervention is most efficient in the low educated group, and least in the medium educated group. This scenario, as well as scenario #2 *(PROMOTE-differentiated)*, would result in a decrease of health inequalities between education groups. These differences between males and females could be explained by the gender-specific distribution across education groups [67], with older females more often falling into the low education group than males. Hence, targeting those with lower levels of education would also affect the health differences between genders.

Unfortunately, we were not able to compare our results with other approaches because to our knowledge no other HIA estimating differential health impacts of physical activity interventions among population subgroups characterised by education has been published.

All in all, the health benefits of all four scenarios from scenario #1 *(PROMOTE-undifferentiated)* through to scenario #4 *(Upward-gradient)* fall far behind the disease cases and deaths that would be avoided under optimal conditions as in scenario #5 *(Equalizing)* or scenario #6 *(Guideline)*. Thus, further research could identify interventions or case studies with greater physical activity changes from the literature, and assess their impact on population health and health inequalities.

### Strengths and limitations

This is the first health impact assessment using the DYNAMO-HIA software tool that has modelled health impacts following behavioural risk factor changes by socioeconomic position. Use of this software as a dynamic model tool for quantitative health impact assessments [56] is a strength of this analysis. A further strength of our analysis is that we used real-world data on intervention effectiveness as well as fictitious scenarios based on published typologies [41, 42, 66].

Limitations stem from model input parameters and assumptions. Specifically, two of our intervention scenarios were based on data from the PROMOTE project, which is an case study of a short intervention implementation within a research project (scenarios #2 and #3). An underlying assumption in our simulation is that the intervention-induced physical activity change observed in this study remains valid over the whole 10-year projection period in our simulation. Otherwise, the IHD, stroke and diabetes cases would adjust to the reference scenario in the long term.

We derived data on physical activity from the GEDA 2014/2015-EHIS dataset, which implemented the EHIS-PAQ to assess physical activity. The EHIS-PAQ provides work-related (including housework and gardening), transport-related, and leisure time physical activity. Unfortunately, work-related activities were not assessed in terms of frequency and duration and therefore could not be used for our analysis. Nonetheless, we believe the GEDA 2014/2015-EHIS provides the best available data on physical activity for Germany.

For our purposes, we categorized physical activity into three groups (insufficiently active, sufficiently active, additionally active) based on the categories used in the WHO recommendations [6]. It is possible that people in our simulation increase their physical activity level in the intervention scenarios without switching to a higher physical activity category. In these cases, the DYNAMO-HIA software tool underestimates health benefits of intervention scenarios compared to the reference-scenario.

We simulated the effect of increased physical activity on three diseases (IHD, stroke and diabetes), but not on cancer. Given that an increase in physical activity of 10 MET-hours per week has previously been shown to induce a 7% reduction in cancer incidence [3], additional health benefits from the reduction of cancer cases can be expected in the intervention scenarios that were not considered in this analysis.

For IHD, stroke and diabetes, the original data source did not provide relative risks differentiated by gender or education [52]. Nevertheless, we used these relative risks since they were also used in the Primetime CE model [68]. For all-cause-mortality, the original data source provided relative risks stratified by potential effect modifiers such as gender and education [53]. We used gender-specific relative risks in this case, but not education-specific relative risks because the confidence intervals of education-specific relative risks were overlapping.

In DYNAMO-HIA, prevalence, incidence and mortality are differentiated by age and gender, but further distinctions (for instance by education or other indicators of socioeconomic position) are not possible. Health disparities between education groups calculated by DYNAMO-HIA are therefore solely driven by differences in physical activity behaviours and can be completely removed by adjusting physical activity levels, which is an oversimplified assumption of reality. Previous research, for example, has found that health behaviours explained only 45% of educational differences in all-cause mortality among men and 38% among women, with physical activity explaining 14 and 9%, respectively [11].

Finally, we used education as an indicator of socioeconomic position. Education seems to be the most frequent indicator of socioeconomic position when examining inequalities in physical activity [69, 70]. Nevertheless, both education and income appear to be important indicators when examining inequalities in physical activity [69]. For instance, leisure-time physical activity has not only been shown to be determined by individual physical activity cognitions and household financial assets, but also environmental and neighbourhood factors. These factors were able to explain both educational and income inequalities in physical activity to a great extent [71]. Even though education and income are correlated indicators, they may explain different causal mechanisms [72].

## Conclusions

The tackling of social inequalities in health is the central challenge of public health [73]. In Germany, these inequalities appear to have increased in recent years [74]. Therefore, health impact assessments with a focus on equity are essential [75]. This paper provides the first assessment of how the overall population health impact varies depending on how intervention-induced physical activity change differs across education groups. The results of this study show that in order to correctly project population health effects and choose between options of intervention types from a public health perspective, data on subgroup-specific intervention effects are needed. Furthermore, this paper highlights the importance of assessing the distribution of health impacts both overall and within a population as interventions with the greatest population health gain might be accompanied by an increase in health inequalities. Further improvements are needed in the analysis and reporting of differential intervention effects across social groups, as well as in methods to estimate population health impacts of interventions that take social inequalities in population health, health determinants and risk estimates into account. Better data on equity impacts of interventions on population health under real-life conditions would help public health professionals and policy makers in designing and implementing interventions suitable for tackling social inequalities in health.

## Data Availability

The datasets used and/or analysed during the current study are available from the corresponding author on request.

## Abbreviations

MVPA: Moderate-to-vigorous physical activity
ISCED: International standard classification of education
IHD: Ischemic heart disease
MET: Metabolic equivalent of task
WHO: World health organization
EHIS: European health interview survey
EHIS-PAQ: European health interview survey-physical activity questionnaire
